# Understanding Alcohol Use Discourse and Stigma Patterns in Perinatal Care on Twitter

**DOI:** 10.3390/healthcare10122375

**Published:** 2022-11-26

**Authors:** Fritz Culp, Yuqi Wu, Dezhi Wu, Yang Ren, Phyllis Raynor, Peiyin Hung, Shan Qiao, Xiaoming Li, Kacey Eichelberger

**Affiliations:** 1 College of Engineering and Computing, University of South Carolina, Columbia, SC 29208, USA; 2 Arnold School of Public Health, University of South Carolina, Columbia, SC 29208, USA; 3 College of Nursing, University of South Carolina, Columbia, SC 29208, USA; 4 Prisma Health Upstate, University of South Carolina School of Medicine Greenville, Greensville, SC 29605, USA

**Keywords:** perinatal alcohol use, thematic content analysis, social media, sentiment, stigma, discourse

## Abstract

(1) Background: perinatal alcohol use generates a variety of health risks. Social media platforms discuss fetal alcohol spectrum disorder (FASD) and other widespread outcomes, providing personalized user-generated content about the perceptions and behaviors related to alcohol use during pregnancy. Data collected from Twitter underscores various narrative structures and sentiments in tweets that reflect large-scale discourses and foster societal stigmas; (2) Methods: We extracted alcohol-related tweets from May 2019 to October 2021 using an official Twitter search API based on a set of keywords provided by our clinical team. Our exploratory study utilized thematic content analysis and inductive qualitative coding methods to analyze user content. Iterative line-by-line coding categorized dynamic descriptive themes from a random sample of 500 tweets; (3) Results: qualitative methods from content analysis revealed underlying patterns among inter-user engagements, outlining individual, interpersonal and population-level stigmas about perinatal alcohol use and negative sentiment towards drinking mothers. As a result, the overall silence surrounding personal experiences with alcohol use during pregnancy suggests an unwillingness and sense of reluctancy from pregnant adults to leverage the platform for support and assistance due to societal stigmas; (4) Conclusions: identifying these discursive factors will facilitate more effective public health programs that take into account specific challenges related to social media networks and develop prevention strategies to help Twitter users struggling with perinatal alcohol use.

## 1. Introduction

Extensive scholarship has discussed health issues related to perinatal alcohol use. Studies link alcohol consumption during pregnancy to poor perinatal outcomes: miscarriage, preterm birth, and low birthweight [1]. Perinatal alcohol use may also induce long-term neurological alterations to the shape and connectivity of the human brain [2,3]. These subsequent lifelong behavioral and cognitive disabilities have been diagnosed as fetal alcohol spectrum disorder (FASD) [1,4,5]. Despite the lack of evidence for any safe threshold of low-level drinking during pregnancy [6], it is estimated that 11.2% of pregnant individuals consume alcohol in North America [7]. Likewise, a recent study in the United States indicated that approximately one in seven pregnant adults had consumed alcohol in the past 30 days and 40% self-reported binge drinking as well [8].

Behavioral patterns and social dynamics have been extensively studied in relation to perinatal alcohol use. Alcohol consumption has been identified as a coping mechanism to help deal with stress, hunger, and poverty [9]. Family dynamics and personal relationships strengthen popular misconceptions that may influence a woman to continue drinking during pregnancy [10]. Moreover, substantial gendered differences regarding perceptions and attitudes about alcohol use have been identified in rural spaces [11]. These findings extend to social and cultural events where pregnant women are similarly encouraged to drink and well-established prenatal health risks are minimized [9,12].

Much of the current literature stems from traditional data housed in national databases [8,13,14]. For example, the Behavioral Risk Factor Surveillance System (BRFSS), which is one of the few national phone surveys, asks willing participants about perinatal alcohol use. The BRFSS survey, however, is limited to self-reported data which frequently relies on simple and straightforward questions [8,13,14]. These inquiries about alcohol use are largely framed as binary situations to help respondents with memory recall issues. The dichotomization of complex behaviors, however, may not accurately reflect the time in which alcohol was consumed during preconception, prenatal, and postpartum periods. Moreover, the data collection process is also subject to several forms of bias. In the same way that certain women may reject to participate in the phone survey, others voluntarily identify themselves as pregnant and willingly answer additional questions. As such, their results might be less representative of other women populations in the United States nor account for metropolitan, rural and regional areas [15]. Overall, these surveys are costly, time-consuming, and susceptible to participant recall bias, socially desirable responding, and the researcher unintentionally biasing participant responses. With the emergence of social media platforms, the accessibility of rich data and reduced costs provide opportunities for novel research at the population level. In contrast to traditional datasets, a person’s ability to project their opinion, independent of national cross-sectional surveys, may reduce memory recall biases [16,17,18]. Moreover, access to real-time data has made the digital landscape synonymous with public discourse, and in certain instances, been viewed as an indicator of public opinion [19,20,21].

The selection of Twitter as the data-source for our investigation is not without its own unique set of challenges and opportunities. In comparison to Facebook and several other social media platforms, Twitter is unique in that it does not collect demographic indicators such as user age, name, race, and gender [22]. Users are able to control their preferred level of personal identification, and if desired, choose to be anonymous on Twitter. While researchers have utilized profile description, account username, and geolocation to estimate gender and ethnicity [23,24], relatively little information is known about the background of most Twitter users. By extension, clandestine user accounts have been referenced in relation to hate speech, online abuse, and sexualization of women’s bodies [20,25,26,27].

Along with these challenges, Twitter offers several advantages. Other studies have emphasized the unique outcomes from account anonymity by investigating how identity concealment has empowered domestic violence survivors to tell their stories [28] and encouraged discussions about mental health issues by providing online solidarity and inter-user support [29]. In comparison to Facebook and other platforms that form social networks from known connections and require verification [30], Twitter users may be less inclined to self-regulate content. While alternative social networks such as Parler—a self-branded free speech alternative to Twitter—provide a unique set of behavioral and attitudinal insights, alt-tech platforms like Parler have become largely associated with the political-right in the United States, and subsequently, may introduce a disproportionate user base for a population level study [31]. Admittedly, Twitter presents its own limitations as well. Content moderation, company policies, user account information requirements, and other elements unique to Twitter undoubtedly generate biases that must be accounted for in our research objectives in order to establish any meaningful insights.

Twitter is relevant to our research—despite its shortcomings—since it frequently appears at the intersection of scholarship dealing with healthcare and population surveillance [32,33,34,35]. In 2021, a scoping review of 755 articles on digital public health surveillance identified Twitter as the most utilized online social network for health communication studies since 2005. It reported the online social network as the most studied platform for research on mental health, communicable diseases, behavioral risk factors, drug utilization, health services, vaccine, and cancer [34]. These novel contributions extend to the realm of Machine Learning as well—underlining Twitter’s role in providing data for substance use detection, disease tracking and forecasting [33,36].

Despite these potential avenues for advancing the current field of research, the role of social networking sites in forming attitudes and altering alcohol consumption patterns in pregnant women remains relatively unknown. Remarkable studies have utilized social media as a tool to extract rich data about the user and substance use [25,26,37], nevertheless, the findings frequently analyze an individual’s social media content in isolation from other users and neglect the influence of other users and ongoing conversations that are unique to each virtual platform. To remedy this scholarly gap within the growing literature, our content analysis utilizes open-source data gathered from Twitter and deploys qualitative methods to illustrate the nuances of tweets that are layered with opinions, experiences, and sentiment. Admittedly, our accumulation of randomized tweets disconnected user-generated content from its original context. Not surprisingly, our study is subject to the same shortcomings as previous social media studies. Despite the constraints from working with such data, we designed research objectives that focus on the ‘social’ component of social networks and draw attention to the discrete patterns that weave seemingly disjointed tweets together to better understand the perinatal alcohol use patterns that are largely absent from the current literature. We argue that a greater emphasis on inter-user exchanges and online discourses will contribute insights about an individual’s (un)willingness to share personal experiences about alcohol use during pregnancy. Furthermore, we assert that a more nuanced discussion about perinatal alcohol use will generate thematic findings about online discourses, underscoring the patterns for sentiment placement within user-generated content and outlining how different narrative positions strengthen online attitudes rooted in societal stigmas.

Given these factors, this exploratory research study sets out to identify communication behaviors, discourses, perceptions, and stigma about perinatal alcohol use from user-generated Twitter content. To undertake this analysis, we must first recognize widespread societal perspectives that perceive alcohol as part of everyday life and view its consumption as a choice [37,38]. In contrast to previous studies that show the emergence of online inter-user support networks for addiction and negative externalities [22,29], our work will illustrate that online solidarity for women dealing with alcohol use during pregnancy is relatively non-existent because societal sentiment and perceptions about alcohol use view abstinence during the gestational period less as a choice and more as a responsibility [39,40,41].

Our study seeks to address the following research questions: 

**RQ1:** What are Twitter users’ posting patterns when they discuss topics and opinions regarding perinatal alcohol use? 

**RQ2:** Is the online social environment on Twitter conducive to helping users leverage the platform to seek professional assistance with drinking during the gestational period?

**RQ3:** Are there any implications we can provide regarding alcohol consumption patterns on Twitter during the COVID-19 pandemic, and in general, in perinatal health under new conditions? 

These qualitative insights may be informative for public health alcohol services, legislation, and perinatal health services as they navigate the evolving trends in perinatal alcohol use and address challenges specific to pregnant adults on Twitter.

## 2. Methodology

### 2.1. Design and Data Collection

In this study, we applied a Twitter Search API V2 [42] as our tweet extraction tool to collect the dataset based on a set of related keywords provided by our clinical team. As shown in Appendix B, we provided a list of sample keywords including primary and secondary keywords, which we used to connect the primary keywords. After the tweet data extraction, we selected a subset of alcohol-related tweets based on the keywords, such as drink, alcohol, booze, drunk, and drank. 

We captured over 6 million original perinatal alcohol related tweets on Twitter ranging from May 2019 to October 2021. By removing ads, spam, pictures, videos, non-English tweets, and those without geolocation disclosure and outside the U.S., the number of related tweets was reduced to 16,134. For this initial study, 500 tweets were randomly selected and coded using qualitative content analysis to discover underlying perinatal alcohol use themes.

### 2.2. Coding Methods

This study operates through the lens of qualitative content and thematic analyses. It begins with an open-coding approach that is data-driven and crystallizes the emerging results by iteratively theming the data [43]. The first coding cycle utilized the ‘descriptive coding’ methodological approach to make sense of the user-generated content by producing generative categories and inventories [44]. The second coding cycle organized disconnected results by using ‘axial coding’ to identify related (sub)categories [45]. Transitioning from coding the data, we iteratively began ‘theming the data’ into specific qualitative thematic areas of the sample of tweets [44]. For instance, the theme for ‘Alcohol Delivery and Consumption during COVID-19’ surfaced from drawing connections between tweets coded to categories related to legislation, consumption behaviors, COVID-19, and political discourse. 

NVivo (Version 12) was utilized to undertake this type of content analysis. The software for qualitative data analysis can be applied to text, audio, video, and multiple forms of media simultaneously. NVivo utilizes ‘parent’ and ‘child’ coding framework to scaffold concepts within complex data sets. It enables researchers with the tools to organize large quantities of (un)structured data in meaningful ways and uncover descriptive insights.

### 2.3. Codebook and Results

Our codebook encompasses several hierarchical levels (see Appendix A). The main level contains eight distinct coding categories: ‘Alcohol use during pregnancy’, ‘Emotions’, ‘Experiences, perceptions, and opinions’, ‘Infant outcomes’, ‘Legislation’, ‘Outcomes aged 0–18’, and ‘Other substances and miscellaneous’. While the focus of our research centers on alcohol related consumption, the final main-level category includes tweets that reference alcohol use in relation to drug and polysubstance use. It also includes references to general categories such as ‘Public Health’, ‘non-English tweets’, and ‘Alcohol advertisements’. 

As noted elsewhere, the most general conceptual ideas are located on the main level of the codebook to help guide the overall research framework of the project [46], meanwhile the underlying categories are inductively and iteratively produced to capture the richness of the data [47]. Beneath the main level of coding themes, several tiers of child codes branch out to capture the nuances generated from line-by-line manual coding. Frequently, these subcodes focus on emotions (visual and descriptive), narrative position and perception (discourse, third person, and first-person); and timeline (prenatal, infant, and long-term).

The Twitter data was imported to NVivo and analyzed by two researchers. Both coders reviewed 30% of the data sample to become aware of the range and the scope of the content. The process entailed frequent meetings to seek consensus when defining qualitative attributes and discuss potential thematic areas. This inductive approach allowed coders to progressively analyze and summarize content into respective themes [46]. Next, the researchers independently coded the remainder of the tweets into corresponding codes and subcodes.

Overall, in this study, two researchers analyzed 500 tweets. Mirroring a previous Twitter content analysis that manually coded 500 tweets [48], our study adopted the same approach to equip researchers with the ability to iteratively process each instance of rich user data and inductively outline the underpinnings for this project and a future project that will deal with the remainder of the data sample. The corresponding codebook includes 12, 292 instances in which researchers referenced coding areas within the passages of text and 518 independent codes. Oftentimes, coding areas overlapped and were coded multiple times to account for explicit and latent meanings. For example, the following tweet captures the textual layers of meaning and complexity of the user-generated data:

*@nytimes Give me no abortion? I’ll give you suicide, a baby with fetal alcohol syndrome, low birth weight, a baby born addicted. When no one adopts this baby, I will give you child abuse, neglect, or abandonment. And where’s my prenatal child support?*

To demonstrate the credibility and trustworthiness of the results, the project resulted in a satisfying Kappa score [49] of 0.836 across the eight independent main-level coding categories (Appendix A). Where differences emerged, the researchers conversed to select the most fitting code.

## 3. Results

Six qualitative themes emerged from the coded Twitter data: (1) Discourses about alcohol use; (2) Perceptions of others’ experiences and behaviors; (3) Personal experiences and behaviors; (4) Family dynamics and intimate relationships; (5) Alcohol delivery and consumption during COVID-19; and (6) Stigma (Table 1). It is important to note that the six thematic areas identified here are different from the main-level codes mentioned in Appendix A and methodological section. The themes listed in Table 1 emerged from iterative coding cycles, and as result, they are more indicative of coding outcomes and less representative of specific codes. 

To better identify perceptions, behaviors, and outcomes, the first three thematic areas were delineated in relation to the narrative position of the tweet. Our textual analysis of the data revealed nuances in self-perception, social stigmas, and a wide variety of value systems. The three interrelated narrative categories appeared throughout the 500-tweet sample with the following frequency: 205 tweets were related to discourses about alcohol use, 170 were related to perceptions of others’ experiences and behaviors, and 158 were related to personal experiences and behaviors. It is important to note that occasionally tweets were double coded to multiple narrative positions due to several factors: ambiguity of narrative voice, lack of context, and rich text saturated with multiple messages [44]. For example, the following tweet was thematically categorized to both discourses and perceptions of others: “You are literally the scum of the earth if you’re consuming alcohol while pregnant.” It is unclear if the brief message utilizes the third-person pronoun ‘you’ to address a specific person in an online exchange or if the tweet’s author is utilizing it as an impersonal subject pronoun to participate in a larger online discussion. For these reasons, it was necessary to apply multiple codes to ambiguous tweets. The other findings —family dynamics and intimate relationships, alcohol delivery and consumption during COVID-19, and stigma—focused on the meanings that related to ways that alcohol use intersects with broader concepts that define society, such as value systems constructed around familial social dynamics and societal transformations onset by the COVID-19 pandemic in 2020.

### 3.1. Discourses about Alcohol Use

Topic-centered tweets assigned to this area engage in broad ongoing conversations, oftentimes interacting with unspecified audiences (*n* = 205). The ‘Discourse’ theme reflects one of the defining attributes of Twitter as a social media platform—which unlike Facebook—fosters virtual interactions among a broad spectrum of known and unknown users [19,29]. Its propensity for scaling multi-user conversations engenders a discursive space and the findings from this thematic area confirm as much.

Subthemes within this category display a wide range of discursive topics trending among users. While several discursive tweets were indicative of favorable attitudes towards alcohol consumption, our thematic analysis showed that most discursive tweets trended towards more negative sentiment in comparison to other narrative positions (Table 2). In particular, any favorable opinions about alcohol consumption were largely overshadowed by discourses related to abortion. Representing 16.1% of all subthemes categorized under ‘Discourses about Alcohol Use’, the ‘Abortion’ subtheme indicates how users associate alcohol use with scenarios related to abortion, and in many instances, captures how online conversations debated if evidence of the former justifies the latter. In short, the patterns from these discussions show that while prenatal alcohol use is closely tied to political topics, it transcends beyond the political realm, illustrating that most Twitter users perceived perinatal alcohol use as harmful to the baby and a violation of motherhood.

Discourses about pregnancy and abortion engaged with ongoing conversations. While it is not entirely clear who the targeted audience is, the tweets clearly engage with outside discussions and ideas. Oftentimes, these virtual interactions encompassed topics related to social movements, legislation, and political tone:

*If she does drugs, drinks one glass of alcohol, takes medicine when she didn’t know she was pregnant, they can charge a murder case against her. Punishable for decades in prison.*

*While this may be accurate, it completely misses the guys point of a mother willfully disregarding the health of the unborn children inside her body by ignoring basic science that says excessive alcohol consumption while pregnant is dangerous? Are we that caught up in feminism?…*

User-generated content in this theme frequently linked abortion to motherhood. The ensuing criticisms about abortion reinforced stigmas related to prenatal alcohol consumption and negative sentiment was a throughline among many of these discursive tweets:

*Drinking alcohol during pregnancy is not harmless! There’s no safe moment or amount of alcohol suggested during pregnancy! #MamáCeroAlcohol #fetalmedicine…*

*…Outrageous! Really not fair! Absolutely inhumane. Who came to this crazy idea? Let’s assume a woman doesn’t know she’s pregnant and uses drugs or alcohol it still isn’t a purpose to end the pregnancy. And if she knows she is expecting and drinks or do drugs: it’s an illness.*

### 3.2. Perceptions of Others’ Experiences and Behaviors

The thematic area titled ‘Perceptions of Others’ experiences and behaviors’ captures opinions and experiences that Twitter users perceive in specific individual other than themselves (*n* = 170). To differentiate from the previous category, these tweets utilize the third person to narrate experiences of others while layering the content with their own personal perceptions and bias. For example, this category would code a tweet describing a specific mother that had consumed alcohol during pregnancy, however, it would not accept a tweet about mothers in general and their prenatal alcohol consumption patterns.

As shown previously, the attitudinal approach to other Twitter users and their descriptions of third parties heavily skewed towards negative sentiment (Table 2). Nevertheless, the category is unique in that it contains a variety of narratives and opinions about alcohol use. Several subcodes reflect behavioral patterns that describe addiction, abuse, coping mechanisms, public behavior, and prenatal alcohol consumption. It also consists of positive behaviors and maternal changes during pregnancy. The recurring theme along the behavioral spectrum is that Twitter users utilized sentiment to emphasize their representation of other persons—either demonizing pregnant adults’ substance use or celebrating their inspirational recovery stories. Most often, tweets deployed strong statements that reflected stigma found at the intersection of prenatal alcohol use and motherhood:

*Her priorities were [obscenity] up since pregnancy. Can’t hold off on alcohol for the sake of her child?!…*

*i gotta friend who’s birth mother gave him up cause she had to choose between retaining custody or going to a court ordered rehab program for drinking…she chose alcohol…*

*…why on earth should a pregnant woman even be taking alcohol? fuess she doesn’t care about the unborn baby.its disheartening cus I know of a girl who constantly takes alcohol while she was pregnant and she lost the baby…*

User-generated content also implied mixed opinions about breastfeeding, nursing, and pumping. These tweets recounted previous experiences and engaged with other Twitter users to discuss different perspectives about drinking alcohol following childbirth:

*To add to the list of strangest things I have seen in a bar: A drunk woman decided to milk herself with her machine. Sorry ma’am but I don’t think alcohol will make the milk better for your baby*

*…You can have a beer! There is sooooo little alcohol that transfers into your milk that you’d have to be falling over drunk before there was enough in there to even affect the baby. The general rule is, as long as you can find and safely hold your baby, you can nurse* *🤗*

*She’s breastfeeding too! Which means the baby is getting alcohol. #LivePD…*

### 3.3. Personal Experiences and Behaviors

‘Personal Experiences and Behaviors’ is a theme that identifies individualized experiences, consumption preferences, and personal opinions regarding alcohol use (*n* = 158). Unlike tweets captured in the prior themes, this content is written in the first-person, explicitly connecting the author to the message. The variety of personalized accounts provide a more unique set of insights and non-conventional opinions regarding alcohol consumption and behaviors. For example, a small subset of tweets projected personal value-systems that either leaned in favor of prenatal alcohol exposure or relayed personal experiences with substance addiction during pregnancy:

*…I’d drink alcohol before I took a newly released pharmaceutical while pregnant. It’s already a semi-touchy subject to begin with, but some physicians forget to remain biased when it comes to playing God.*

*…I abused drugs and alcohol for years on &amp; off--really nasty ones that do terrible damage to a fetus. Poor nutrition, no medical care, &amp; could not stop using even when I suspected I was pregnant.*

It is important to note that of the 158 tweets attributed to first-person experiences, the two examples listed above were the only tweets from the sample which expressed interest or self-identified the user with perinatal alcohol use. Meanwhile, among tweets written in the narrative third person, the ‘Perceptions of Others’ category captures these same dynamics, documenting a substantial increase in references perinatal substance use (25 of 170 tweets). To further underscore the divide between ‘Personal Experiences and Behaviors’ and other narrative positions, users emphasized positive emotions to tell their own stories (66.9% non-negative descriptive sentiment). Subthemes gravitated to narratives about abstinence, withdrawal, and healthy behaviors. In particular, one subtheme focused on tweets related to positive maternal changes during pregnancy:

*I have reached a new level of pregnancy tired. I call it: sans-alcohol hangover* *🤪🥱 basically I’ve been awake since 3 am. Guess this is my life now…*

*I guess being pregnant 2/3 of the last 3 years really took a tool on my taste for alcohol I feel like I’m 13 again*

However, not all sentiment was positive within the category. Themes from elsewhere in the codebook appeared here as well—specifically the topic of abortion:

*…I’m pro-choice and I’d cuss a woman out who was planning to carry a baby to term yet drinking alcohol*

*…I just can’t imagine how decestated I would be personally to find out I had been unkowingly harming my baby. Whether it be pills/alcohol/illicit drugs babies aren’t intended to be exposed to any of it and if that was me I would want to consider abortion*

Despite the prevailing negative attitudes toward abortion, users provided more personalized tweets that widened the conversation about the emotionally charged subject. In ways that were non-existent elsewhere, individuals authored tweets that connected alcohol to sexual activity. From pleasure to coercion, more nuanced messages identified alcohol as a driving force for sexual encounters, and at times, a catalyst for inception:

*if i become pregnant it’s gonna be tonight because brad just got a haircut and alcohol is gonna be involved*

*should I give up alcohol or my birth control* *😮 …*

*It’s a lot of you grown ass men on here tweeting about how disgusting rape is but have either tried to [obscenity] w me and my friends when we were 16 or used alcohol to get a girl in bed w you…*

To appreciate the diversity of this thematic area, it is necessary to cross-reference it with the other narrative thematic areas. While individuals were more willing to self-identify with past substance use and addiction, it is clear that Twitter users were extremely hesitant to disclose personal ongoing issues related to perinatal alcohol use. As previous studies on social media platforms have indicated, women face disproportionate levels of hate speech, online vitriol, and trolling—leading individuals to self-censor and delete highly maligned tweets [25,26,50]. Thus, it stands to reason that these factors paired with external societal stigmas and negative discursive sentiments offer several likely explanations for the absence of personal experiences about alcohol use during pregnancy.

### 3.4. Family Dynamics and Intimate Relationships

Tweets describing alcohol use shared meaningful information about familial and personal social relationships (*n* = 169). Social media content in this area routinely outlined how familial realities are defined and altered by alcohol consumption. In contrast to prior thematic areas, this category surfaced throughout several categories in the codebook: Infant outcomes, Outcomes 0–18, Discourse, Other’s experiences and behaviors, and Personal experiences and behaviors. Its frequent occurrences helped narrate different familial nuances and depict the differences between the gestational, infant and childhood stages. While the objective of our study focuses on the perinatal period, user-generated content required that we acknowledge the scope and lasting impacts from alcohol consumption during pregnancy:

*…I have a niece who cannot function on her own. She has fetal alcohol syndrome, FAS, and was adopted as a baby. Here are no facilities for this in Florida. She will probably spend her later years in a special section they are beginning to put in prisons for this reason. So sad.*

*…we had a whole family. But then toxic ppl and alcohol drove him away. Crazy not even strongest drug could take me away form my kids.*

In addition to impacts on familial structures, tweets identified (inter)generational family members’ role in driving alcohol consumption. One example occurs in social gatherings and events, a coding area which was heavily influenced by family dynamics, traditions, and holidays (Table 3):

*I have 3 weeks to get my alcohol tolerance back so my family doesn’t kill me at my Grandmas 75th birthday*

*…I grew up in a family with many members addicted to alcohol—though thankfully some found sobriety -- and it persists down through the generations.*

*…Under the current rules I can identify as such…Grandmother was born there…Plus Irish on my Grandfather’s side…Alcohol is water to me…*

In the same way that prior generations influenced consumption patterns, anticipation for the next generation shifted attitudinal and behavioral habits as well. One subcode in particular, ‘Parenthood and parenting strategies,’ captured these dynamics by focusing on how adults perceived, managed, and used alcohol within their respective parenting roles (*n* = 39). In response to the belief that prenatal alcohol use harms babies, parents exhibited the following approaches:

*Our child’s environment and the behaviour they see their role models in regards to alcohol is more than we think…*

*…I don’t smoke &amp; seldom drink alcohol. It impairs my judgement. Cigarettes &amp; alcohol are expensive. You have to launder clothing more often which wears them out prematurely plus they cause health problems in kids…*

Humor and non-negative sentiments appeared adjacent to these forms of parental awareness. It underscores the burden of parenting children, suggesting that alcohol serves as a coping mechanism of sorts. The tweets displayed below contain insufficient background to contextualize the message with either humor or seriousness, however, it is clear that alcohol use is associated with the stresses of parenting.

*It costs over $235,000 for parents to raise a child today. And that’s just for the alcohol…*

*#FakeLEGOFacts 9 out of 10 parents consume copius amounts of alcohol while their child is busy with LEGO’s.*

### 3.5. Alcohol Delivery and Consumption during COVID-19

User-generated content retweeted commercial ads about alcohol delivery services and discussed recent changes in their purchasing behavior and consumption (*n* = 75). Tweets commented about temporary changes to alcohol purchasing legislation in the following states: Louisiana, Maryland, Massachusetts, Missouri, New York, and Texas. The vast majority of tweets were reshared from commercial accounts and lacked additional textual contributions, however, a subset of Twitter users voiced their approval about the emergence of remote alcohol purchasing:

*Restaurants and bars are getting real creative to sell alcohol through carry out and delivery and I wholeheartedly appreciate it*

*…I just ordered alcohol delivery so things are looking up*

*Currently have margaritas being delivered to my home. We need to make alcohol delivery and take out a permanent thing moving forward…*

Endorsements for new purchasing pathways were also met with criticism. A small minority group of users questioned how less regulated purchasing might foster domestic violence, underage drinking, and in general, increase consumption volume and frequency during widespread quarantines at the beginning of the COVID-19 pandemic in 2020:

*…I have seen a lot of concern for domestic abuse victims who are trapped at home with their abusers, and I’m glad someone is thinking of them. Is anyone concerned that bars are still being allowed to trade alcohol by delivery?*

*…Hi Karyn. Discussion this afternoon on alcohol. Domestic violence in the context of alcohol home delivery, off sales designated essential, no supermarket purchase limits was much in folk’s minds. Let’s keep in touch on this…*

It is important to note that no tweets in our randomly selected sample of 500 tweets explicitly mentioned consuming alcohol during both the pandemic and gestational stage. In several instances, individuals on Twitter commented about increased consumption and efforts to abstain from alcohol, nevertheless, these infrequent mentions were unrelated to pregnancy.

### 3.6. Stigma

The stigmatization of perinatal alcohol use is among the least defined thematic findings in our codebook, nevertheless, it is perhaps the most pervasive concept found throughout the user data set (*n* = 179). At times, societal stigmas occupy the central idea of user-generated content. In other moments, variations of stigma more subtly frame the ways that ideas and opinions are expressed. Between these two ends of the spectrum, the pervasiveness of perinatal related stigmas produces profound contextual barriers for pregnant adults and deeply influences the decision-making process when seeking health services for screenings, recovery from alcohol addiction, and additional services to other substances [41,51]. To accurately define perinatal stigmas, our analysis outlines three distinct subthemes: *Individual*, *Interpersonal*, and *Population Level*.

The first area explores the ways that critical societal perceptions may motivate women to delay, or even, deny themselves access to health care services. Accounting for external pressures is important because it reveals the additional barriers that pregnant women must confront to initiate and maintain access to vital health services:

*A patient confided that she has been coping w/ the pandemic thru heavy alcohol use, + is ready for treatment. Between her work (a nurse), a death in the family during this time, and the stress of it all, she can’t cope…*

*if they could speak up and admit there mistakes and try being better people if alcohol is the problem than maybe they should think of quitting*

The individualization of the gestational period not only makes the mother solely responsible for the well-being of the fetus, but it also reinforces ongoing societal stigmas about motherhood and further isolates the pregnant women that struggle with disparaged behaviors. Moreover, this widespread attitudinal position—which views abstinence during pregnancy as a simple ‘choice’—ultimately reduces alcohol to a non-addictive substance and leads pregnant women to believe that their actions are easily rectifiable, and therefore, do not warrant healthcare and professional recovery services.

Additionally, the interpersonal subtheme looks to deal with perinatal alcohol use challenges that emerge among family, friends, and social networks. The perception from close associates on Twitter suggests that perinatal alcohol use is indicative of less-than-ideal parenting—both in the present and in the future.

*…I’m a drug user when it comes to alcohol and weed (the legal and socially acceptable drugs). I’ve lost family and friends to opiates and have seen first hand how criminalizing the issue has made it more dangerous and harmful to those involved.*

*One parent and an alcohol addiction from the other spiraled my family out of control…*

*…My father left my mother with 4 kids under the age of 5 (she was an immigrant w no family here) no support, saw him maybe 6 times, alcohol over parenting…*

Unlike the prior subtheme on individual, this type of stigmatization deals with the entire family. It views alcohol use as a destructive force on interpersonal relationships and utilizes user experiences as testaments to warn of the consequences that are correlated with alcohol consumption.

The concept of stigma appears most clearly within the final subcategory. The ‘Population Level’ thematic space is rooted in more tangible examples given that societal norms and values crystallize in the form of laws and public safety policies. If the notion that pregnant women dealing substance use are unlikely to utilize perinatal health services appears either irrational or unfounded, the following examples give credence to their concerns and contextualize how the popular perceptions give life to societal stigmas:

*…It’s legally required to maintain a 0.08 blood alcohol level through the duration of your pregnancy in Alabama, and if they find out you went to another state to take a break, they abort the baby and send you to prison for murder*

*They justified it because they said they smelled alcohol and it was endangering a child. But he was sober for 7 years. 2010 officer came to our school with cps and interviewed us…*

As shown above, the apparatus of public services and legislation generates fear and mistrust. The formalization of negative perceptions validates stigmas mentioned elsewhere, especially when the outcomes skew towards punishment and regulation. It also undercuts the establishment of public health services and challenges their baseline objectives designed to address perinatal alcohol use in meaningful ways.

## 4. Discussion

The principal findings from our qualitative content analysis revealed patterns about how people utilize Twitter to carry out discussions about perinatal alcohol use on the online platform. It demonstrates that most tweets either concentrated on expressing opinions about other persons or engaged in broader alcohol-related online discourses (Table 1). The prevalence of negative sentiment and critical attitudes about prenatal alcohol use also reinforced prevalent social stigmas, offering several explanations as to why Twitter users were unlikely to share their own personal experiences. Additionally, the emergence of alcohol delivery services during the pandemic appeared from user endorsements, highlighting recent developments in the way that individuals purchase and consume alcohol. With pregnant adults both virtually isolated (hate speech, misogyny, societal stigmas, etc.) and physically isolated (i.e., social distancing, quarantine, etc.), our research findings emphasize the need to continue monitoring prenatal alcohol consumption in an environment with fewer purchasing restrictions and advocates for the expansion of health services that consider prevention and intervention strategies on social media platforms.

### 4.1. Twitter Posting Patterns on Perinatal Alcohol Use (RQ1)

The thematic analysis of social media content in our study is consistent with previous qualitative studies in this field of research [19,28,29]. It illustrates the ways that people talk about perinatal alcohol use, share opinions [52,53,54], and captures how Twitter users overwhelmingly deploy negative sentiment when representing the experiences of individuals (60.3% negative vs. 39.7% non-negative). Given the nature of semi-anonymous character of Twitter accounts, which oftentimes permit emotionally charged language and hate speech [55], we found that generalized conversations about perinatal alcohol consumption were layered with aggressive, and at times, violent language. As a whole, these online discourses frequently viewed prenatal alcohol use as a violation of motherhood and blatant disregard for the baby.

The toxicity of Twitter engagements carries profound implications for users struggling with alcohol use during pregnancy. Contrary to the social structure of Facebook, which allows users to build support networks through personal connections and private groups [56], Twitter funnels trending topics and tweets into public conversations, and consequently, exposes the user to a much broader audience. These dynamics have deep implications for users dealing with substance use. A difference exists between a plea for help that receives no attention and one that goes viral for the wrong reasons [50]. For example, in contrast to more receptive online attitudes that embrace tweets from individuals struggling with depression and gendered violence [25,50], our study shows that prenatal alcohol consumption is critically perceived as a choice, and as such, abstinence during pregnancy is viewed as the mother’s responsibility. Our thematic analysis confirms as much, discovering that just 2 out of 158 tweets written in the narrative first-person openly acknowledged prenatal alcohol use. While it remains unclear if toxic inter-user dynamics motivate pregnant adults to seek support through more personal and private social media channels, our results displayed an unwillingness to tweet about ongoing personal substance issues and the silence implies a certain degree of fearfulness of being subjected to online bullying.

### 4.2. Twitter Toxicity: Stigma, Silence, and Intervention (RQ2)

In a much broader sense, the scale and banality of toxic online engagements demonstrates the strong connection between societal stigmas and perinatal alcohol use. The stigmatization of women dealing with substance issues not only discourages mothers from utilizing important health services [41,51], it generates detrimental internal self-stigmas that exacerbate alcohol use during pregnancy [41,51,57,58]. By understanding these dynamics at play, our study emphasizes the need to address those most at-risk, specifically Twitter users that would seek to leverage the platform for assistance. It also acknowledges that the identification of at-risk mothers is insufficient and recognizes that healthcare and social services lack certain features to make meaningful changes. To address the fallout from stigmatizing perinatal alcohol use, healthcare and social services must undertake the endeavor of (re)educating the larger population about the risks of alcohol use during pregnancy and seek to remove external criticisms that are associated with perinatal substance use. Furthermore, healthcare institutions must also recognize its complicity in societal stigmas by focusing less on policing substance use in the pregnant population and increasing support services for housing, parenting, nutrition, trauma, and domestic violence [41].

### 4.3. COVID-19: Alcohol Delivery Service, Isolation, and Shifting Consumption Patterns (RQ3) 

Our thematic analysis also underscores opinions that may be indicative of consumption alterations during the COVID-19 pandemic and associated various state alcohol policies. Researchers have already identified factors such as depression, income loss, and living alone as contributors to increased alcohol consumption during the pandemic [59,60,61,62]. Meanwhile, other work has found little to no evidence that perinatal alcohol use rates have increased during COVID-19 pandemic, findings which point towards reductions in social gatherings and social distancing measures [63,64]. While these findings about drinking during the pandemic have been mixed, the impacts from temporarily lifting alcohol purchasing restrictions remains unknown for pregnant adults. Our qualitative approach addressed this gap in the research by identifying attitudinal approval among users and significant interest in alcohol delivery in the United States. In particular, it found positive sentiment in the following states mentioned by users: Louisiana, Maryland, Massachusetts, Missouri, New York, and Texas. Meanwhile, in the state of Mississippi where delivery service is not allowed, one Twitter account shared the following: “today is that day I wished Mississippi had an alcohol delivery service.” Thus, with pregnant adults utilizing alcohol and other substances to cope with boredom, lack of schedule, and stress [65], it is important to monitor the rising demand for alcohol delivery services. Our content analysis illustrates this phenomenon clearly. From May of 2019 to the beginning of the global pandemic in March 2020, our textual inquiry only identified 14 tweets related to alcohol delivery service. In the months following, however, user-generated content gravitated towards the same coding theme in 59 tweets. While it is still unclear how access to alcohol delivery service has affected drinking in domestic spaces during social distancing and quarantine periods, Twitter users textually demonstrated its popularity, oftentimes describing changes in their consumption habits—or at the very least—declaring a willingness to adopt new consumption patterns in a less restricted purchasing environment.

In conclusion, these findings help elevate the awareness of perinatal drinking perceptions and promote large-scale changes that will lessen the disproportionate burdens placed upon pregnant women. Attention to these results will directly impact at-risk women and at-risk babies. The ramifications from this call to action do not rest solely upon women’s shoulders, but extend to social service organizations, the medical community, policy makers, funders, and society as a whole.

## 5. Study Limitations

Several limitations challenge research on perinatal alcohol use moving forward. First, the user-generated data for our content analysis is highly subjective and shaped by personal perceptions. Unlike a survey or semi-structured interviews, our random selected tweets were not guided by a set of research questions, and thus, encompass a wide variety of messages about alcohol use. Second, only 500 tweets were coded from the larger sample size. It is anticipated that future work will build upon these results and analyze the remaining tweets. Third, as mentioned previously, the anonymity of Twitter accounts provides little personal information about the users studied. Drawing certain conclusions were unfeasible due to the lack of information about age, occupation, race, sex, and socioeconomic status. Access to more information about users’ backgrounds would allow researchers to parse the latent and explicit meanings more accurately within complex messages. Fourth, the sample data of user-generated content in this study is inherently disconnected from the original context in which the tweets were created. Despite our emphasis on the importance of inter-user exchanges, our current analysis of individual tweets solely focused on fragmented online conversations, and at times, was not able to grasp the potential influence of the adjacent tweets, comments, and additional dynamics of the online environment. Finally, it is likely that the data collected from Twitter is incomplete. With the same freedom that users articulate attitudes and opinions, users can strategically self-regulate their own accounts [25]. It is unclear if negative online exchanges—hate speech, bullying, and misogyny—influenced users to silence their experiences by deleting their own tweets.

## 6. Future Research

Inter-user interaction is an important issue for future research. To develop deeper insights, further work should explore segments of online interactions by focusing on Twitter threads (a series of connected tweets from the same user), mentions (a tweet that references another username, e.g., @username), and retweets (re-posting another user’s tweet). Among other examples, a sentiment analysis of Twitter mentions or an evaluation of retweet behaviors may provide meaningful findings to this growing field of research. Our next phase of the project development plan will further investigate inter-user posting patterns and retweet behaviors associated with alcohol use communications among the perinatal population.

Relatively little scholarship on perinatal alcohol use has commented on the intersection of race and rurality. It has been well established that rural spaces present a significant obstacle to public health and access to prenatal health care [11,66,67] and that women living in rural spaces experience greater degrees of depression, abuse, and neonatal morbidity [66,68,69,70]. Research has also highlighted specific challenges that are unique to racial and ethnic groups of women and children [67,71]. Most notably, these studies range from clinical work with Mexican Americans [72] to research about American Indian and Alaska Native populations [73,74]. While these remarkable studies have mentioned rurality and race individually, the intersection of race and space has yet to appear as the central focus of perinatal health studies that deal with alcohol use. In the same way that we view thematic content analysis and artificial intelligence as a meaningful instrument for social media perinatal alcohol use risk-prevention, the same combination presents an alternative avenue to reach marginalized communities that oftentimes exist beyond the limits of healthcare services.

As noted earlier, the COVID-19 pandemic created disproportionate impacts across different sectors of society. The physical isolation from the pandemic has further encouraged studies on the development and accessibility of telehealth services [75,76]. In the same way that technological advancements provided new healthcare opportunities for pregnant women in a period defined by social distancing, our study shows that users are leveraging the same access to the internet to consume alcohol at home. The resulting health impacts remain unclear. Thus, a deeper analysis about alcohol delivery services might provide insights on how less-regulated purchasing measures have affected consumption patterns, rates of domestic violence, perinatal health outcomes in states with new legislation, and rural-urban public health objectives to assist women that are most susceptible to prenatal alcohol intake [77,78].

## 7. Conclusions

In this study we analyzed shifting behaviors, attitudes, and perceptions about perinatal alcohol use with Twitter data. These qualitative results are relevant to the fields of public and perinatal health, content analysis, and social media intervention. It is evident that perinatal alcohol use remains prevalent in society and that an increased focus in policy and research will be required to improve these circumstances in society. Overall, the combination of both qualitative methods and quantitative methods in this project helped underscore the different degrees in which users subjectively shape ideas and share values about alcohol consumption among pregnant adults.

The findings of this study hold significant implications for the future of public health. In the same way that social stigma and toxic discourses motivate users to self-regulate their experiences by removing personal tweets, the thematic areas of our study outline a conceptual groundwork for forthcoming prevention and intervention strategies. As shown elsewhere, social media provides accessible data and artificial intelligence transforms it into actionable items for health screening and patient diagnosis [79,80,81]. Artificial Intelligence advancements stemming from user generated data are also being utilized to address depression, child maltreatment, domestic violence, firearm violence, and suicide [80,81,82,83,84,85]. Thus, it stands to reason that our qualitative themes, paired with natural language processing and artificial intelligence [83,84], would carry significant implications for both the mother and baby. Targeting vanishing tweets and disseminating resources directly to at-risk users would alleviate pressures onset by deep social stigmas and provide public health policy makers with novel information to improve initiatives and assist healthcare practitioners with efforts to increase access to perinatal health services in rural and marginalized communities.

## Figures and Tables

**Table 1:** Alcohol Use and Pregnancy Themes and Descriptions.

Theme	Description	Sample Tweet	Number of Tweets
Discourses about Alcohol Use	Content addressing general ideas, non-specific audience, and engagement with broad societal conversations	*They’re whole argument is that they feel they need to protect the unborn. The MOTHER is the one who is responsible for the unborn. She is the one who needs to stay healthy and avoid alcohol…*	205
Perceptions of Others’ Experiences and Behaviors	Third-person narrative that expresses opinions about the experiences and behaviors others	*…Or the girl I knew who gave birth to three drug/alcohol babies. N dumped them on her mother in law…*	170
Personal Experiences and Behaviors	First-person narrative to describe personal experiences and behaviors	*If I wasn’t pregnant I’d probably be in the hospital with alcohol poisoning again* *😂*	158
Family Dynamics and Intimate Relationships	Content describing familial and personal social relationships	*I knew a girl suffering from severe alcohol abuse come to find out she was raped by her father got pregnant…*	169
Alcohol Delivery and Consumption during COVID-19	Legislation, advertisements, and alcohol delivery in the US	*…I ordered an embarrassing amount of alcohol delivery today.*	75
Stigma	Common negative perceptions and attitudes toward perinatal alcohol use	*…If you can’t give up alcohol for a pregnancy, don’t have children. You’re going to be a [obscenity] parent…*	179

**Table 2:** Frequency of Descriptive Sentiments in Relation to Narrative Position.

	Negative Descriptive Sentiments	Example	Non-Negative Descriptive Sentiments	Example
Discourses about Alcohol Use	75.9%	*if you think it’s okay to even have a sip of alcohol during your pregnancy, you DO NOT deserve to be a mom*	24.1%	*They say a lot of people die because of Alcohol Bt they never realize how many of them are born because of it* *😂😂*
Perceptions of Others’ Experiences and Behaviors	60.3%	*…One pregnant lady and her two dim witted companions. 9 cans of alcohol. No shoes. Talking the whole time. Laughed at literal quiet emotional scenes. Talked [obscenity] at me as they left because I dared stare at them during their rude, classless behavior*	39.7%	*…I went over to my wife and asked if she really wanted a mint julep and she goes…it has alcohol in it Michael. I know this but do you want one? Haha I forget she’s #pregnant! #dadbrain #familytime #horses #KyDerby #funny*
Personal Experiences and Behaviors	33.1%	*I spent the next 2+ years doing everything I could to validate how [obscenity] I felt inside. I stole money, drugs, alcohol from my family. I lied about anything and everything…*	66.9%	*Two years ago today i gave up a relationship with my father because he decided a relationship with alcohol was more important than one with his child. I’m still proud of myself for that*

**Table 3:** Social gatherings, events, and locales.

Event	Number of Tweets Referenced (*n* = 52)
Family gatherings	8
Birthday party	4
Baby showers	4
Bar	4
Wedding and marriage events	2
Sporting events	2
Afternoon Tea	1
LGBTQ gathering	1
Nightclub	1
Thanksgiving	1

## Data Availability

The data presented in this study are available on request from the corresponding author.

## Appendix A. NVivo Codebook for Perinatal Alcohol Use Tweets

**Main-Level Coding Categories and Second-Level Subcategories**	**Number of Tweets**
A_During pregnancy	52
- Behaviors, habits, and patterns	33
- Outcomes	22
- Outside recommendations and advice	18
- Prenatal care	3
A_Emotion	128
- Descriptive emotions	98
- Visual emotions (emoji’s)	56
A_Experiences, perceptions, and opinions	373
- Discourse	205
- Other’s experiences and behaviors (outside perceptions)	170
- Personal experiences and behaviors	158
- Social drinking (who)	16
- Social gathering, events, locales (where)	35
A_Infant outcomes	41
- Breastfeeding, nursing, and pumping	4
- Diets that include alcohol	1
- Family dynamics and relations	16
- Fetal Alcohol Spectrum Disorders	18
- Foster Care and Adoption	11
- Negative health outcomes	24
- Prenatal care and child services	11
- Race	1
- SIDs and fatal outcomes	1
A_Legislation	42
- Drinking age	2
- Drinking and driving	5
- ID	3
- Laws for selling alcohol (specific to vendors)	14
- Legal issues, and crimes	4
- Police and law enforcement	8
- Prison	1
- Prohibition	5
- Regulation of Alcohol Advertisement	2
- State laws	4
A_Outcomes aged 0–18	36
- Abuse, abandonment, neglect, trauma	6
- Adoption	4
- Autism	1
- Child Custody	2
- Children’s environment of alcohol	4
- Family dynamics and intimate relationships	10
- Fetal Alcohol Spectrum Disorders	4
- Genetic and hereditary	4
- Negative health outcomes	14
- Physical Appearance	1
- Violence and crime	2
A_Purchasing	15
- Being ID’d and carded for purchasing alcohol	2
- Being perceived by others while purchasing alcohol	3
- Financial cost	4
- Legislation or regulation of purchasing age	1
- Stocking up	1
- Underage drinking	3
Other substances and miscellaneous	251
- Alcohol miscellaneous	101
- Drug use	72
- Non-English language tweets	23
- Polysubstance use	62
- Public health	88
Note: A—Alcohol.	

## Appendix B. Samples of Twitter Searching Keywords

**Primary Keywords**
Alcohol, Cocaine, Amphetamines, methamphetamine, Hallucinogens, nicotine, Opioid, sedatives, diazepam, Tobacco, SUD, Heroin, Cannabis, MDMA, LSD, weed, meth, XTC, benzodiazepines, stimulants, Morphine, Fentanyl, Codeine, drug, drug use
**Secondary Keywords** - Perinatal Related childbearing, expecting, prenatal, Birth weight, pregnant woman, baby, infant, pregnancy, babyloss, baby loss, pregnancy loss, iam1in4, waveoflight, Pregnant, Preggers, Baby time, Childbirth, Giving birth, Newborn, Delivery, Abortion, Homebirth, Prenatal class, Miscarriage, Fetal, Stillbirth, Stillborn, Preterm, Postterm, Due Date, Baby loss, Neonatal, Neonatal Intensive Care, Neonatal Intensive Care Unit, Infant loss, Low birth weight, LBW
- COVID-19 Related: CORONA, corona, COVID-19, covid 19, covid, coronavirus, Coronavirus, Corona Virus, NCOV, sarscov2, sars cov2, c2019ncov, n95, ppeinfluenza
- Disorder Related: Communication, Risky health behaviors, Inadequate nervous system, Memory issue, Poor behavior, outcome, rural, policy-relevant knowledge, Nutrition, feelings of anxiety, defect, abnormality, disorder, down syndrome, addiction, addict, racial disparity
